# Letter to the Editor: Peer Assessment Rating (PAR) scoring of cleft patients treated within a regional cleft centre in the United Kingdom

**DOI:** 10.1177/14653125221106219

**Published:** 2022-08-26

**Authors:** Haydn Bellardie

Dear Sir,

I read the article by Furness et al. (2022) with great interest. The authors have managed to highlight the increased difficulty of orthodontic treatment of patients born with a cleft and have also highlighted several limitations of such a retrospective study.

For instance, it is difficult to tell from the paper whether any records were missing, what level of training any of the operators had or the distribution of cases from each unit. Were any of the cases treated by a trainee or non-cleft specialist orthodontists?

The outcomes are presented combining all cleft types. This approach, while providing an overall figure for the region or unit, does tend to cloud the actual outcome for cases of unilateral (UCLP) and bilateral cleft lip and palate (BCLP). These two groups present with their own different dental anomalies and growth patterns, which are very different from other cleft types. They therefore should be looked at separately. In addition, one has to take care interpreting outcomes as the small numbers in such a study can add to inaccuracies.

Of greater importance, this paper highlights the differing outcome figures from different centres, when compared to those reported by Deacon et al. (2007) and Stonehouse-Smith et al. (2022). For cases of UCLP, the Peer Assessment Rating (PAR) improvements are in the range of 56%–84%, and for cases of BCLP 53%–80%. These figures suggest that further investigation is required to explain the large differences in these outcomes, whether they be due to surgical protocols, surgeon skills or orthodontist skills.

I would like to suggest that when comparing unit or centre outcomes that Nomogram and PAR outcomes are presented as bar charts with the traffic light approach, similar to the way GOSLON outcomes are presented.

**Figure 1.**
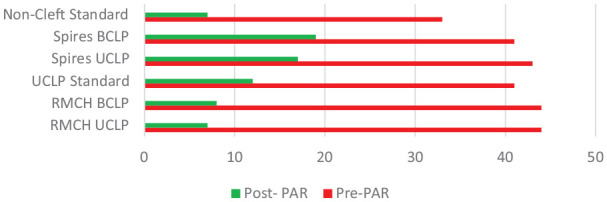
Mean pre- and post-treatment PAR scores.

The mean pre-and post-treatment PAR Scores (Figure 1) illustrate the figures from different centres or units. The pre-treatment PAR scores above 40 confirm the increased difficulty of cases of UCLP and BCLP.

**Figure 2.**
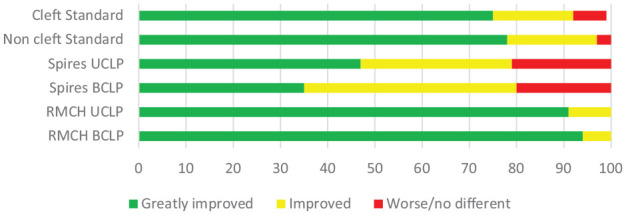
Nomogram % as a bar chart.

Figure 2 illustrates the percentage changes from different units and the National Cleft Standard.

The figures from Royal Manchester Children’s Hospital, considering the results of a high-volume operator, suggest that perhaps the time has come to re-visit the Cleft Standard.

The Cleft Collective is in a perfect position to consider a serious prospective investigation of Orthodontic Outcomes and to establish more up-to-date recommendations for orthodontic care in the Cleft Service. I would encourage them to embark on such a study as their five-year-old cohort are approaching the age of 12 years and are likely to shortly be embarking on orthodontic treatment.


Haydn Bellardie University of the Western Cape, Cape Town, South Africa

